# Hsp90α and cell death in cancers: a review

**DOI:** 10.1007/s12672-024-01021-0

**Published:** 2024-05-10

**Authors:** Bin Liu, Daohai Qian

**Affiliations:** Department of Hepatopancreatobiliary Surgery, The First Affiliated Hospital of Wannan Medical College, Wuhu, 240001 Anhui China

**Keywords:** Heat shock protein 90α, Cell death, Signaling pathways, Cancer therapeutics

## Abstract

Heat shock protein 90α (Hsp90α), an important molecular chaperone, plays a crucial role in regulating the activity of various intracellular signaling pathways and maintaining the stability of various signaling transduction proteins. In cancer, the expression level of Hsp90α is often significantly upregulated and is recognized as one of the key factors in cancer cell survival and proliferation. Cell death can help achieve numerous purposes, such as preventing aging, removing damaged or infected cells, facilitating embryonic development and tissue repair, and modulating immune response. The expression of Hsp90α is closely associated with specific modes of cell death including apoptosis, necrotic apoptosis, and autophagy-dependent cell death, etc. This review discusses the new results on the relationship between expression of Hsp90α and cell death in cancer. Hsp90α is frequently overexpressed in cancer and promotes cancer cell growth, survival, and resistance to treatment by regulating cell death, rendering it a promising target for cancer therapy.

## Introduction

As a molecular chaperone for cellular proteins, heat shock protein (Hsp) is a highly conserved protein. It is essential for preserving normal cell development and survival. Previous studies have shown that Hsp is upregulated in various types of tumors, which is strongly associated with resistance to tumor therapy [1]. Hsp can be generically categorized as Hsp90, Hsp70, Hsp60, and tiny heat shock proteins (15–30 kDa) based on their molecular weight. Interestingly, the *Hsp90AA1* codes for Hsp90α, which is involved in the invasion and growth of cancer cells. It is produced extracellularly [2].

Cell death is a basic physiological process that occurs in all living organisms. It is involved in immune response, aging, organ preservation, autoimmunity, and embryonic development. Recent studies significantly advanced our understanding of the mechanisms underlying cell death. Particularly, these studies focused on the roles of immunity and internal environment homeostasis. This study aimed to provide a comprehensive overview of the role of Hsp90α in cell death and its significance in the development and treatment of cancer. Through a comprehensive review of relevant literature, we mainly focused on the regulatory mechanisms of Hsp90α in various types of cell death, signaling pathways, and its mechanisms of action in the biological characterization and treatment of cancer.

## Hsp90α structure and function and Hsp90α inhibitors

HSP90 is a highly conserved molecular chaperone with four isoforms, including Hsp90α and Hsp90β in the cytoplasm, glucose-regulated protein 94 (Grp94) in the endoplasmic reticulum, and tumor necrosis factor receptor-associated protein 1 (Trap-1) in the mitochondria. They all share a common structural organization [3]. Hsp90α is an extensively studied isoform of Hsp90 in mammalian cells, which is a stress-inducible form of Hsp90. There are several heat shock elements (HSEs) located upstream of the *Hsp90α*. Heat shock factor 1 (HSF1), a major transcriptional regulator of the heat shock response, binds to the *Hsp90α* and promotes the expression of Hsp90α [4]. Specifically, the HSE at positions − 96/− 60 enhances the expression of Hsp90α, while the simultaneous presence of the HSE complex at positions − 1031/− 1022 is required for heat shock induction. This allows timely expression of Hsp90α by HSF1.

Hsp90α is primarily found in the cytoplasm and facilitates the folding and assembly of guest proteins through its three-dimensional structure [3, 5]. It consists of three functional regions: the amino-terminal (N-terminus), the central region, and the carboxy-terminal (C-terminus) [6]. The N-terminus of Hsp90α is crucial for its function, which possesses an adenosine triphosphate (ATP)-binding site and ATPase activity. Binding and hydrolyzing ATP can regulate the structure and function of Hsp90α. The central region contains multiple repetitive tetratricopeptide repeat (TPR) units, which interact with other proteins to form complexes and mediate signal transduction. The C-terminus of Hsp90α contains a Met-Glu-Glu-Val-Asp (MEEVD) sequence, which is an important structural domain for interacting with other proteins [6–8]. Many proteins associated with cellular signaling can bind to the C-terminus of Hsp90α, such as protein kinase B, p53, epidermal growth factor receptor, etc. [3, 9–11]. Hsp90α works in conjunction with auxiliary proteins, such as Hsp70, Hop, and p23, to promote the correct folding and assembly of guest proteins [5]. These client proteins include various signaling proteins, receptors, kinases, and transcription factors, which play important roles in cell division, apoptosis, and cell cycle progression. Hsp90α is also involved in the regulation of numerous cell signaling pathways. Several key proteins in these pathways interact with Hsp90α. By binding to and regulating these signaling proteins, Hsp90α can modulate physiological and pathological processes, including cell proliferation, differentiation, apoptosis, and metabolism. Some of these signaling pathways include Raf/MEK/ERK [12], PI3K/Akt [13], and NF-κB [14]. Furthermore, Hsp90α is induced in stress conditions. Cell exposure to external stimuli, like heat shock, oxidative stress, or drugs, increases the expression of Hsp90α, which helps protect cells against damage. Specifically, heat shock stimulating factor activation promotes stress-induced expression of Hsp90α [4].

Hsp90α has been shown to be overexpressed in many cancers and is associated with malignant cell survival, proliferation and metastasis, making it one of the most important targets for anticancer drug discovery.Hsp90α inhibitors inhibit Hsp90α by inducing degradation of the substrate protein through conformational changes caused by binding to the appropriate regulatory sites. These include N-terminal inhibitors, C-terminal inhibitors, and inhibitors that interfere with the binding of Hsp90 co-chaperones. Researchers have been searching for effective Hsp90α inhibitors. Some of the known Hsp90α inhibitors include geldanamycin and its derivatives, 17-AAG (17-allylamino-17-demethoxygeldanamycin) and other compounds synthesized subsequently, and ganetespib. These compounds have shown antitumor activity in in vitro and in vivo studies, and some have entered clinical trials.

## Cell death

Cell death is an important event in the cellular life cycle. There are various modes of cell death, including apoptosis, autophagy-dependent cell death, necroptosis, pyroptosis, cuproptosis, ferroptosis, and disulfidptosis, etc. Apoptosis and necrosis are the two predominant forms of cell death [15]. Apoptosis is a tightly regulated programmed cell death that does not release inflammatory cytokines, while necrosis is an unexpected, unregulated, unscheduled passive death that releases inflammatory cytokines. However, more and more studies have shown that necrosis is also closely regulated by signal pathways, called regulatory necrosis, including necroptosis, ferroptosis and so on [16]. The cells in the human body are always in the process of constant renewal, which is accompanied by the death of primordial cells, some of them die through natural aging, and some die through non-aging. If cell death is regulated by genes, it is “programmed”, also known as programmed cell death. Such type of programmed cell death is vital in maintaining tissue and organ homeostasis and function while preventing uncontrolled cell proliferation [15].

## Hsp90α and cell death

**Fig. 1:**
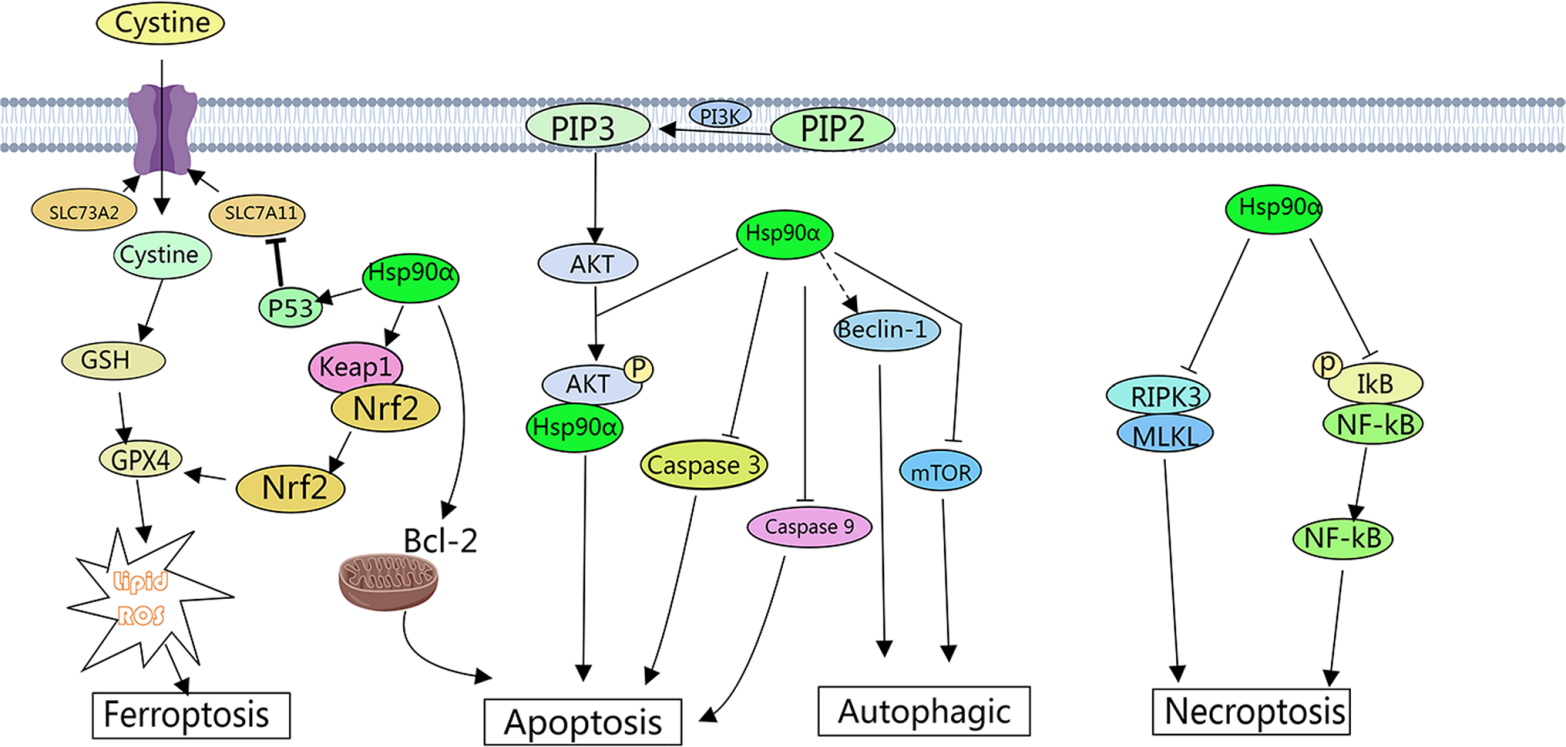
The involvement of Hsp90α in cell death pathways

Hsp90α plays a crucial role in regulating various cellular processes and signaling pathways, such as cell proliferation, apoptosis, senescence, and the stress response [13, 17, 18]. Hsp90α is also closely associated with different types of cell death (Fig. 1).

## Hsp90α and apoptosis

Apoptosis is a process of active cell death under the control of genes. It is usually triggered by the activation of internal signal pathways, such as cytokines, receptor-mediated signal transduction and intracellular signal pathways. The Bcl-2 family of proteins includes a major group of regulatory factors associated with apoptosis [19]. In particular, the interaction between Hsp90α and Bcl-2 regulates mitochondrial permeability and promotes or inhibits apoptosis [20]. Simultaneously, Hsp90α also binds to apoptosis activators like caspase-3 and caspase-9 in their non-apoptotic state, inhibiting their function [21, 22]. Moreover, the cell membrane enzyme PI3K converts phosphatidylinositol diphosphate (PIP2) to phosphatidylinositol triphosphate (PIP3), activating the intracellular protein Akt. After binding to Akt, Hsp90α maintains its structure and stability and promotes its phosphorylation and activation to regulate apoptosis [23–25].

Studies have demonstrated the critical role of Hsp90α in various scenarios. For instance, in pancreatic ductal adenocarcinoma cells, the Hsp90α inhibitor Octyl Gallate enhanced apoptosis, hindered endothelial-mesenchymal transition-induced M2 macrophage polarization, suppressed Hsp90α secretion, and inhibited tumor growth [26]. Similarly, deoxyelephantopin, a diterpene lactone derived from the saffron thistle plant, induced mitochondrial apoptosis in hepatocellular carcinoma (HCC) cells by targeting Hsp90α [27]. The Hsp90α inhibitor kW-2478 dampened the malignant behavior of tumor cells induced by BCR/ABL and markedly reversed indirubinib resistance [24]. Gold nanoparticles can also help tumor therapy through Hsp90α. Multi-branched gold nanocomposites possess a plasmonic resonance effect and facilitate gene photothermal combination therapy through CRISPR-Cas9 delivery, thereby promoting tumor cell apoptosis [28]. Multifunctional gold nanorods can induce tumor cell apoptosis via low-temperature photothermal interactions and suppress tumor growth via RNA interference [29]. These findings indicate a strong association between Hsp90α and apoptosis.

## Hsp90α and necroptosis

Necrosis is an unprogrammed form of cell death that typically occurs in response to strong external stimuli, severe injury, or disruption of the intracellular environment. However, necroptosis is highly regulated. Unlike apoptosis, it is usually triggered by dysfunction of apoptosis inhibitory proteins, and when the apoptotic pathway is inhibited, the cell can undergo “selective” cell death through necroptosis. Necroptosis is an active process dependent on intracellular signal transduction, triggered by corresponding ligands by activating death receptors. Hsp90α plays an important regulatory role in necroptosis. At present, it is believed that the occurrence and regulation of necroptosis are mainly related to proteins such as tumor necrosis factor (TNF-α), Caspase-8, receptor interacting protein kinase 1 (RIPK1), receptor interacting protein kinase 3 (RIPK3) and mixed kinase region-like (MLKL) protein, etc. [30]. Hsp90α can regulate the process of necroptosis by affecting the stability of RIPK3 and MLKL [31]. Studies have shown that Hsp90α can bind to activated RIPK3, preventing its binding to the MLKL protein. This interaction inhibits neuronal cell necrosis after trauma by modulating the RIPK3 pathway [31, 32]. In pancreatic β-cells, inflammatory stress triggers the release of Hsp90α, which can regulate cell necrosis [33]. Under hypoxic conditions, Hsp90α mediates the drug resistance of sorafenib to human hepatocellular carcinoma mainly by inhibiting necroptosis [34]. In addition, inhibition of Hsp90α can protect cultured neurons from necroptosis induced by hypoxia and glucose deprivation by reducing the expression of RIP3 [35]. Furthermore, Hsp90α can also affect necroptosis by regulating cell signaling pathways. IKK, an important kinase responsible for the phosphorylation and degradation of IκB, has been found to play a key role in necroptosis [36]. IκB phosphorylation leads to the release of active NF-κB, which participates in cellular necrosis. Hsp90α can regulate IKK activity and influence the phosphorylation of IκB [36, 37].

## Hsp90α and autophagy-dependent cell death

Autophagy is the process by which damaged, denatured, or senescent proteins and organelles are transported to lysosomes. Under the regulation of autophagy-related genes, lysosomes digest and degrade these proteins and organelles. Excessive autophagy in cells results in autophagy-dependent cell death. Autophagy-dependent cell death is a type of programmed cell death that depends on the autophagy mechanism or its components, which belongs to type II programmed cell death [38]. Hsp90α can affect the expression and stability/activity of signaling proteins such as Beclin1, Ulk1, LAMP-2A, and Akt [39]. Additionally, Hsp90α can modulate autophagy by mediating mTOR signaling, a crucial negative regulator of autophagy [40–42]. Moreover, inhibiting Hsp90α enhances temozolomide-induced autophagy-dependent cell death [43].

## Hsp90α and ferroptosis

Ferroptosis is a distinct form of cell death that differs from apoptosis and necrosis [44]. It is mainly caused by iron overaccumulation and reactive oxygen species-dependent accumulation of lipid peroxides [45]. Ferroptosis can be biologically and chemically induced. Chemical inhibition of the extrinsic cystine/glutamate antiporter system xc− or the intrinsic glutathione peroxidase 4 (GPX4) is the classical method to trigger ferroptosis [46]. The Xc− system comprises solute carrier family 7 member 11 (SLC7A11) and solute carrier family 3 member 2 (SLC3A2) subunits. The expression and activity of SLC7A11 are negatively regulated by *TP53* [47]. In normal conditions, Nrf2 is bound to Keap1 in the cytoplasm, which inhibits its transcriptional activity. However, under oxidative stress, Nrf2 is released from Keap1 sequestration and translocates to the nucleus, upregulating the expression of the target *GPX4* [48]. Hsp90α also induces Acsl4-dependent ferroptosis in gliomas by dephosphorylating the serine 637 site of dynamin-related protein 1 [49]. Additionally, a natural compound known as Timosaponin AIII can promote ferroptosis in non-small cell lung cancer by targeting Hsp90-mediated ubiquitination and degradation of GPX4 [50]. In acute kidney injury, Legumain promotes tubular ferroptosis by facilitating molecular chaperone-mediated autophagy of GPX4 [51]. In conclusion, there is a relationship between Hsp90α and ferroptosis. Hsp90α can affect ferroptosis by regulating the stability and function of GPX4 and interacting with proteins involved in iron metabolism.

## The role of Hsp90α in cancer

Hsp90α plays a crucial role in the development and progression of cancer. As a molecular chaperone, it regulates the stability and function of multiple apoptosis-related proteins, thereby exerting anti-apoptotic effects [52]. By protecting cancer cells against stress-induced apoptosis, Hsp90α promotes their survival and proliferation [52]. Additionally, Hsp90α regulates various cancer-related signaling pathways and proliferation-linked proteins. It activates cell-cycle regulatory proteins and upregulates the expression of receptors for cell proliferation, contributing to cancer cell proliferation and growth [23, 53, 54]. Moreover, Hsp90α regulates the stability and function of cancer-associated proteins, including transcription factors and matrix metalloproteinases, enhancing cancer cell invasion and metastasis [55, 56]. Recognized as a potential therapeutic target, Hsp90α provides new opportunities for treating cancer [57]. Hsp90α inhibitors have demonstrated anticancer activity in clinical trials. These small molecule inhibitors disrupt protein folding and stability in cancer cells by inhibiting Hsp90α activity, leading to cancer cell apoptosis [58–60]. On the other hand, Hsp90α agonists were shown to enhance Hsp90α activity, increasing the sensitivity of cancer cells to treatment and improving the efficacy of other anticancer drugs [61].

## Hsp90α and hepatocellular carcinoma

Hsp90α plays a crucial role in hepatocellular carcinoma (HCC) and is closely related to its development and progression. Previous studies have revealed that Hsp90α-dependent Bcl-2-related transcription factor 1 promotes cell proliferation in HCC by regulating the stability of c-Myc proto-oncogene (c-MYC) mRNA [20]. This suggests a significant association between Hsp90α and HCC development and cell proliferation. It was also found that deoxynivalenol, a 44-sesquiterpene lactone isolated from Elephantopus scaber Linn was able to induce mitochondrial apoptosis of HCC cells in vitro and in vivo by targeting Hsp90α [27, 62]. Another compound named 8u inhibited the invasion and metastasis of HCC cells by inhibiting Hsp90α and the PI3K/Akt signaling pathways [23]. Additionally, the expression of Hsp90α has been linked to the chemoresistance of HCC cells [34]. Hsp90α can also be a plasma biomarker for the diagnosis of HCC [63].

## Hsp90α and gastric cancer

It has been discovered that Hsp90α overexpression is closely associated with the development of gastric cancer (GC) and lymph node metastasis [64]. Comparing the overall 5-year survival of GC patients with the level of Hsp90α expression, patients with low levels of Hsp90α expression had better 5-year overall survival [65]. Additionally, Hsp90α plays a pivotal role in the early diagnosis of GC and cancer cell invasion. Studies have reported significantly higher serum levels of Hsp90α in patients with GC compared to the control group, with a sensitivity of 52.50% and a specificity of 92.50% [66]. Furthermore, it has been observed that lnc-CTSLP4 can recruit the E3 ubiquitin ligase ZFP91 and regulate HNRNPAB-mediated Snail transcription by interacting with Hsp90α. This interaction effectively inhibits the metastatic potential of gastric cancer cells [67].

## Hsp90α and breast cancer

Hsp90α plays a critical role in the development and progression of breast cancer. Abnormal plasma levels of Hsp90α have been linked to the development of breast cancer [63]. ION-31a, a derivative of alkaloids, can target Hsp90α and inhibit metastasis and angiogenesis in breast cancer [68]. Similarly, other alkaloid-derived compounds can hinder breast cancer metastasis and angiogenesis by targeting Hsp90α [69]. Furthermore, Hsp90α plays a crucial role in lymphangiogenesis and lymph node metastasis in breast cancer. It has been discovered that extracellular Hsp90α promotes lymphangiogenesis and lymph node metastasis in tumors [70]. Additionally, Hsp90α synergizes with clusterin to facilitate epithelial-mesenchymal transition and metastasis of breast cancer. Low-density lipoprotein receptor-related protein 1 (LRP1) is involved in this phenomenon [71]. Moreover, the long-chain non-coding RNA NKILA has been found to regulate the expression of Hsp90α, NF-κB, and β-catenin in breast cancer cells [72, 73]. These findings suggest that NKILA may play a crucial regulatory role in the development and progression of breast cancer. The specific survival of patients with high Hsp90α expression was significantly shorter than that of patients with low Hsp90α expression. In addition, patients with high Hsp90α expression had significantly shorter distant metastasis-free survival [74, 75]. These findings underscore the importance of Hsp90α in the development, progression, and prognosis of breast cancer.

## Hsp90α and pancreatic cancer

Hsp90α promotes metastasis and chemoresistance in pancreatic cancer through its receptor LRP1 [76]. This suggests that Hsp90α/LRP1 signaling plays a crucial role in the malignant transformation and treatment resistance of pancreatic cancer. Additionally, Hsp90α has been implicated in the development of pancreatic cancer [77]. It has been found that myeloid-derived macrophages and secreted Hsp90α both contribute to the development of pancreatic ductal adenocarcinoma. Studies have confirmed the therapeutic potential of targeting Hsp90α expression in pancreatic endocrine tumors [78]. Furthermore, inhibitors of Hsp90α have shown therapeutic effects in pancreatic cancer. NVP-AUY922 induces IGF-1Rβ degradation through catalyst-mediated autophagy and exhibits pro-apoptotic effects on pancreatic cancer cells [79]. Additionally, certain compounds, such as peptides derived from bovine hemoglobin and octyl gallate, target secreted Hsp90α and inhibit pancreatic cancer metastasis and growth [26, 80, 81]. These findings suggest the therapeutic potential of Hsp90α inhibition in pancreatic cancer.

## Hsp90α and other cancers

Furthermore, Hsp90α has demonstrated a close association with various types of cancers and holds potential applications in cancer diagnosis and treatment. Studies have shown that the plasma level of Hsp90α in patients with nasopharyngeal carcinoma can be used not only as a diagnostic indicator, but also as a predictor of prognosis [82, 83]. Hsp90α also shows promise in the diagnosis of other cancers, such as cervical cancer and lung cancer. Plasma Hsp90α combined with squamous cell carcinoma antigen testing in cervical cancer patients increases the sensitivity and specificity of cervical cancer diagnosis [84]. Serum concentrations of Hsp90α were significantly increased in patients with thymic carcinomas, thymomas, thymic neuroendocrine tumors and non-thymomatous myasthenia gravis compared to patients who underwent thymectomy revealing regular thymic morphology or controls [85]. Moreover, elevated plasma expression of Hsp90α has been linked to poor chemotherapy efficacy and prognosis in small cell lung cancer. The mechanism of which may be related to Hsp90α by attenuating the Akt/GSK3β/β-catenin signaling pathway [86].

Hsp90α plays a significant role in the development and progression of various cancers, including lung cancer, melanoma, and colorectal cancer. Overexpression of Hsp90α in lung cancer has been shown to increase stem cell-like and metastatic behavior and promote metastasis and invasion by regulating the Hsp90α/uPA/MMP2 signaling pathway [87]. The plasma level of Hsp90α also predicts the clinical outcome of lung cancer [88, 89]. Hsp90α induces the differentiation of immunosuppressive myeloid cells in melanoma through the TLR4 signaling pathway [90]. Furthermore, Hsp90α expression level can help the diagnosis and predict the prognosis of melanoma [91]. Hsp90α expression is associated with tumor metastasis and poor prognosis in colorectal cancer [92, 93]. By modulating the NF-kappa B signaling pathway, Hsp90α promotes the migration and invasion of colorectal cancer cells [94]. Additionally, Hsp90α induces colorectal cancer cell invasion through CD91/LRP-1 and NF-kappa B-mediated expression of integrin αV [95].

**Fig. 2:**
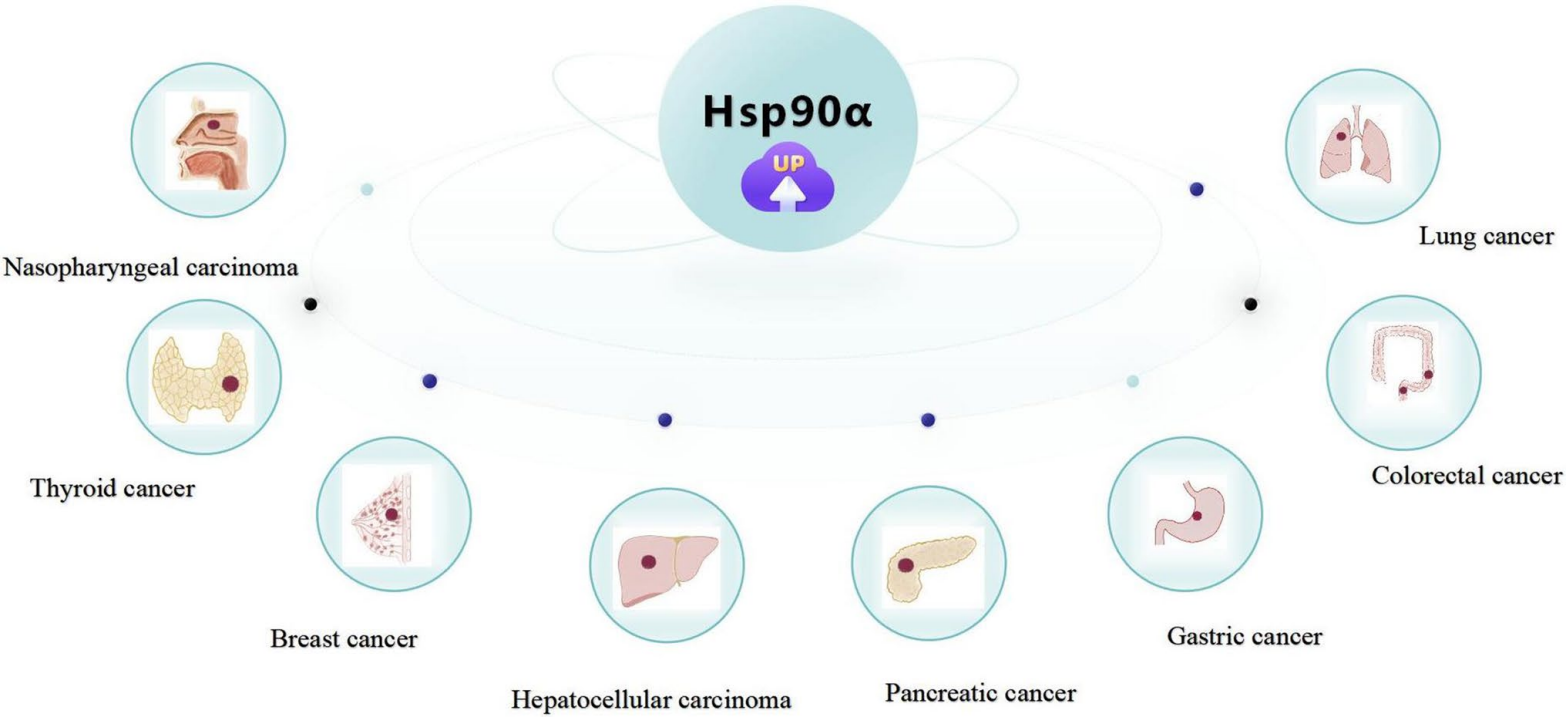
Increased expression of Hsp90α in various cancers

Hsp90α also plays a crucial role in other types of cancers, such as head and neck cancer and oral cancer. Studies indicate that Hsp90α is correlated with the clinical manifestations of head and neck cancer and oral cancer by regulating tumor cell infiltration and M2 polarization of macrophages [96, 97]. The expression of Hsp90α reduces exosome-driven malignant behavior and M2 polarization of macrophages in oral cancer through triple silencing of CDC37, Hsp90α, and Hsp90β [97]. Moreover, the serum level of Hsp90α is a potential biomarker for oral squamous carcinoma [98] (Fig. 2).

## Conclusion

Hsp90α holds a crucial regulatory role in cell death. The expression level of Hsp90α is commonly elevated in cancer and linked to tumor progression and drug resistance [63, 76]. To further advance its application in cancer treatment and personalized medicine, future studies should delve deeper into the molecular mechanisms of Hsp90α in cancer and its interactions with other signaling pathways. Hsp90α inhibitors still confront challenges and limitations in clinical research, including drug resistance and side effects. Consequently, comprehensive studies are necessary to optimize the use of Hsp90α as an anticancer target for amplifying its potential application in cancer treatment and individualized medicine.

## Data Availability

Not applicable.
